# 

**Published:** 1910-10-29

**Authors:** 


Telegraphic Address: Ospedale, London.

THE MODERN MEDICAL AND INSTITUTIONAL JOURNAL.

Telephone Number: 2734 Gerrard.

NEW SERIES. No. 194, Vol. VIII. No. 1266, Vol. XLIX.

SATURDAY, OCT. 29TH, 1910.

PRICE THREEPENCE

